# Characterization of fluid in facial sinuses on post-mortem CT in case of death by drowning

**DOI:** 10.1007/s00414-025-03493-3

**Published:** 2025-04-11

**Authors:** Lucia Fernandes Mendes, Leonor Pedreira Lago, Coraline Egger, Jérôme Schmid

**Affiliations:** 1 Department of Radiology and Interventional Radiology, Lausanne University Hospital and Lausanne University, Lausanne, Switzerland; 2 Lausanne-Geneva, University Center of Legal Medicine, University of Geneva, Geneva, Switzerland; 3 Geneva University Hospital and University of Geneva, Geneva, Switzerland; 4 Geneva School of Health Sciences (HEdS GE), University of Applied Sciences and Arts Western, Geneva, Switzerland

**Keywords:** Post-mortem computed tomography, Post-mortem imaging, Diagnosis, Drowning, Paranasal sinuses

## Abstract

Recent statistics show that drowning deaths are a reality in Switzerland. Although drowning remains a diagnosis by exclusion for forensic pathologists, post-mortem multidetector computed tomography (PMCT) is a real complementary resource to establish the cause of death. The aim of this study was to determine whether the sinuses’ fluids visualized in post-mortem MDCT in case of drowning have specific characteristics that can be related to the submersion process and provide an additional element to the diagnosis of death by drowning. A few studies exist on the subject, but none of them compared putrefied bodies and drowned cases. A balanced dataset was retrospectively collected of 108 cases of natural death, putrefied bodies and drowned cases at the University Center of Legal Medicine in Geneva. For each paranasal cavity, the fluid and sinus were segmented in the slice with largest liquid quantity to derive liquid-to-sinus surface ratio (LR), and mean density (MD) in Hounsfield units (HU) of the fluid when present. For all sinuses, the MD was significantly different between putrefied and drowned groups. The average LR was statistically different for frontal and maxillary sinuses. Using cut-off values as Youden indices from ROC curves, promising specificities (Sp) and sensibilities (Se) were obtained, using single (e.g., frontal sinus: LR cut-off = 0.15: Sp = 76%, Se = 68%; MD cut-off = 44,55HU: Sp = 93%, Se = 64%, or maxillary sinus: LR cut-off = 0.14: Sp = 56%, Se = 86%; MD cut-off = 34,91HU, Sp = 65%, Se = 85%) or all (logistic regression: Sp = 80%, Se = 92.6%) measurements. This study identified potential leads for discrimination of drowning cases from natural deaths and putrefied bodies.

## Introduction

Cases of death by drowning are a reality in Switzerland, with an average of 45 deaths by drowning each year as reported by the Swiss Rescue Society [1]. To establish this type of observation, it is necessary to be able to recognize and establish a diagnosis of drowning. However, drowning is a diagnosis by exclusion. That is, it can only be made after eliminating other possible causes of death. In addition, determination of a post-mortem diagnosis is sometimes difficult to make, in particular depending on the state of conservation of the body. Indeed, it can be in a state of advanced cadaveric alteration or found in an environment that leads to intracorporeal changes, thus making autopsies more complex [2]. Computed tomography (CT), regularly performed in clinical practice and introduced in forensic medicine, is then presented as a real resource when establishing the cause of death. It therefore seems relevant to focus on the potential of imaging in the specific context of drowning.

Zivkovic and al. [3] conducted a study to examine and compare the presence and amount of free fluid in the sphenoid sinuses between drowning cases without signs of putrefaction and those with post-mortem signs of putrefaction. They also analyzed the presence and amount of free fluid in the sphenoid sinuses of bodies found indoors and showing signs of putrefaction. Indeed, it is known that a certain amount of fluid can be found post-mortem in the sinuses, due to tissue liquefaction. They extracted the liquid from the sinuses with a syringe. According to their findings, immersion was the primary cause of presence of fluid in the sphenoid sinuses, not putrefaction, meaning that a specific amount of free fluid in the sphenoid sinus can be recognized as a significant predictor. However, for putrefied bodies recovered from water, the presence of free fluid in the sinuses does not directly imply that drowning was the cause of death. A similar study from Heo et al. [4] did not find a statistically difference and a strong correlation between the measurement of sphenoid sinus fluid volume in drowning cases using PMCT and autopsy. They propose the utilization of PMCT with volumetric analysis prior to autopsy in cases of drowning.

Many studies have also used postmortem CT imaging to assess the presence and amount of fluid in the paranasal sinuses. A state of the art regarding the diagnosis of drowning using postmortem CT was published in 2014 [5]. It showed that the presence of fluid in paranasal sinuses, among other parameters, is determinant in the drowning’s diagnosis. In other studies, liquid density was also calculated using Region of Interest (ROI) by measuring Hounsfield Units (HU). Vander Plaetsen and al., with a 32-slice multi-detector CT scanner (Aquillion 32, Toshiba Medical systems and a 64-slice multi-detector CT scanner (Discovery CT750HD, General Electric Medical Systems) [6], pointed out that up to 98% of subjects who drowned had fluid in the maxillary and ethmoid sinuses, 88% in the sphenoid sinuses and 83% in the frontal sinuses. None of the cases belonging to the control group showed fluid in the paranasal sinuses, which represents a significant difference (for all sinuses, *p* < 0.001 except for the frontal sinus, *p* < 0.05). This study therefore demonstrates that the presence of fluid in the sinuses can support the cause of death by drowning. Christe and al. [7] also observed significant differences. Images were performed on a GE Lightspeed QX/I unit (General Electric). Axial slices were acquired with a collimation of 4 mm × 1.25 mm. Indeed, all drowning cases presented fluid in the paranasal sinuses, particularly in the maxillary and sphenoid sinuses. This last point is also corroborated by the more recent study of Kawasumi and al. [8], which demonstrated that the accumulation of fluid in the maxillary and sphenoid sinuses was significantly associated with drowning. It should be noted that in the control cases (not drowned), the presence of liquid was much less frequent, but was still present.

Mishima and al. [9] also used PMCT, 64-row CT scanner (Somatom Definition AS; Siemens Healthcare, Forchheim, Germany) with the following parameters: 120 kV; quality reference, 400 mAs; thickness, 64 × 0.6 mm; and field of view, 500 mm, to obtain quantitative data from certain organs of the respiratory system (lungs, trachea, sinuses), digestive system (stomach, duodenum) and thoracic cavity (heart, diaphragm). The results showed significant differences in three parameters: the distance between the lungs, the volume of the stomach and the density of the gastric contents. However, no significant difference was found in the paranasal sinuses between the cases of drowning and natural death.

In 2013, Kawasumi and al. [10] published a complementary study with the aim of evaluating the qualitative difference in fluid accumulation between drowning and non-drowning cases. An 8-channel multi-detector row CT scanner (Aquilion 8; Toshiba Medical Systems, Tokyo, Japan) was used for all examinations. In all cases, CT was performed with two scans. First, the head was subject to conventional CT with the following parameters: 120kVp; the tube current was changed arbitrarily based on the installed mode; and collimations, 4.0 and 8.0 mm. The second scan was from the head to the pelvis in helical mode with the following parameters: 120kVp; the tube current was changed arbitrarily based on the installed mode; and beam pitch, 0.875. The reconstructed slice thickness of the images was 2.0 mm. Based on the analysis of the receiver operating characteristic (ROC) curve for chosen characteristics (e.g., volume in ml or average UH of the liquid), threshold values were computed to separate the groups of drowning and natural deaths. According to this study, the threshold values chosen in the ROC analysis, characterized by the points on the ROC curve closest to the origin (0, 1), were 1.03 ml and 27.5 HU. Similarly, Youden indices [11] were 1.03 ml and 37.8 HU [8].

The PMCT performed before the classic autopsy is therefore a visualization and documentation tool which, in the light of the different results obtained, could be useful in the diagnosis of death by drowning. Another potential sign of drowning visible on post-mortem CT (PMCT) can be found in the stomach. This is known as Wydler’s sign. It is characterized by a three-phase sedimentation of gastric contents: gastric contents, water, and foam [12] Many parameters have previously been studied and, in view of the particularly promising results obtained by the analysis of the paranasal sinuses, it is this track that was chosen to explore in the context of this work.

The objective of this study is therefore to provide an additional element to the diagnosis of death by drowning. To do this, two hypotheses will be tested by comparing the quantity and density of liquid in three groups: drowning cases, natural death cases and altered body cases. To achieve this, the feasibility and performance of a radiological protocol will be tested to quantify and measure the density of the liquid present in the sinuses using different measurements derived from the CT images.

## Method

In cases of death from natural causes, it is assumed that there will be very little or no fluid present in the sinuses. Thus, first hypothesis is that there is a difference in the amount of fluid between cases of natural death and cases of death by drowning. Then, the presence of liquid in the sinuses for altered bodies being proven, second hypothesis is that the quantity of liquid and/or the radiological density of the liquid makes it possible to differentiate a process of drowning from an altered body.

In order to answer the question posed, a diagnostic pilot study has been carried out, based on the semi-quantitative analysis of the fluid present in the sinuses using PMCT examinations.

### Study design and ethics

This study supported a thesis in the Master of Science HES⁠-⁠SO - UNIL in Health Sciences and was approved by the Cantonal Ethics Commission for Research on Human Beings (CCER) in Geneva on July 2021 (Project ID: 2021 − 00966).

The cases were selected from the database available at the University Center of Legal Medicine (CURML), Geneva site. A 64-slice multi-detector CT scanner (LightSpeed VCT, General Electric, Milwaukee, United States) was used with the following parameters: 120 kV; 400 mAs; rotation, 1 s; slice thickness, 1.25 mm and a ScanFov, Head. The date of death was located between the year 2016, date of commissioning of the scanner, and 2020. In total, 36 cases of death by drowning were identified. All cases of death by drowning were included in the study, regardless of their radiological alteration index (RAI) [13].

**Fig. 1:**
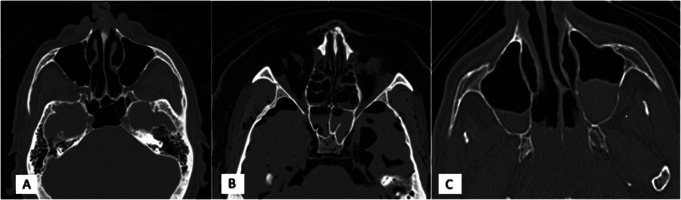
Illustration of the three groups: natural death (**A**), putrefied body (**B**) and drowned case (**C**) as shown in Osirix software on a multiplanar reconstruction, axial slice and bone window

The control sample was composed of 36 cases whose cause of death is natural, linked to a cardiovascular pathology. Only adult cases with a RAI of less than 20 were included, in order to prevent the body from being in a state of advanced alteration. The third group, consisting of 36 decomposed bodies, was selected based on their RAI. The latter had to be equal to 100, so that the state of alteration of the bodies was maximum and comparable. In total, 108 cases were therefore analyzed. The Fig. 1 illustrates the three groups.

### Data collection

**Fig. 2:**
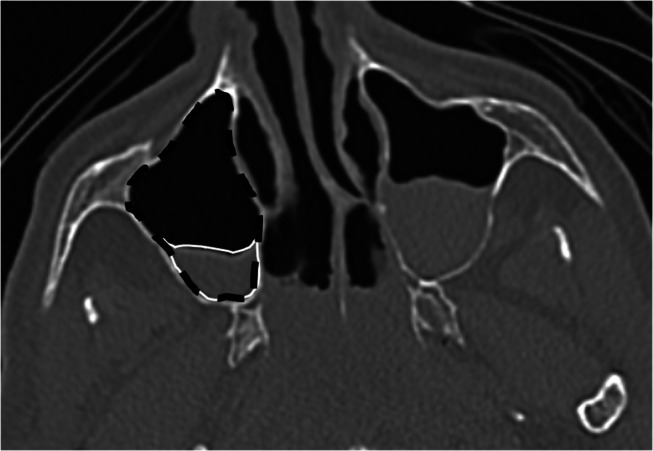
Example of fluid (white) and sinus (black) segmentation in PMCT image from a drowning case with a RAI = 0, where the fluid surface is maximal, using Osirix software on a multiplanar reconstruction, axial slice and bone window

In order to assess the quantity of liquid and characterize it from a radiological point of view, various measurements were taken using a radiological viewer OsiriX (OsiriX DICOM Viewer MD [Software]. Version 13.0.1, Switzerland, https://www.osirix-viewer.com). For each sinus and the liquid present, two different measurements were carried out:

1. Areas of contoured fluid and sinus. These contours were manually drawn on the slice of the CT volume in which the amount of liquid was the largest. Based on the areas of the fluid and sinus we were able to derive the liquid-to-sinus surface ratio (LR) (Fig. 2).

2. Density and standard deviation in HU of the liquid from regions of interest (ROI) defined as small circles. To consider the inhomogeneity of the liquid, multiple ROI were used instead of a standard radiological ROI. In order to determine the number of ROI to place in each sinus, we relied on the LR to estimate the number of circular ROI to place. The defined rule was:

- LR ≤ 1/3: use of 1 to 3 ROI, depending on the available quantity of the liquid.
- LR > 1/3: use of 5 ROI.

Using these different density measurements in each ROI, an average of the densities as well as a standard deviation of the mean densities were computed for each sinus.

All values were input with the EpiData software (EpiData Software, v.4.6.0.6, 2021, Danemark, http://www.epidata.dk) to avoid entry errors according to a case report form (CRF) validated by the Ethics committee. They were then exported and listed in an Excel table to facilitate calculations and interactions with the statistical software used. Data collection was carried out according to the same criteria and in the same way for all groups. Only cases with mucosal thickening, or absence of fluid or even sinuses, had missing data.

### Statistical analysis

Forty-four variables were defined. STATA 16.0 software (StataCorp, College Station, Texas, United States) was used for the various statistical analyzes with a significance threshold set at α = 0.05. Normality tests indicated that the variables did not follow a normal distribution. Consequently, the choice of statistical tests fell on non-parametric tests. For the comparison of surface area ratios and liquid density, the analysis of variance (ANOVA) test was not applicable for the three groups. Indeed, the conditions of application were not met. So, the Wilcoxon-Mann-Whitney test was used to compare the groups two by two (drowning/natural and drowning/altered).

Then, ROC curves were created with the aim of determining threshold values. An analysis was carried out to determine the threshold values of the surface area or liquid density ratios between the natural death group and the decomposed bodies as well as the Youden index. The threshold value corresponding to the maximum Youden index (sensitivity + specificity − 1) was also defined. We based ourselves on the most optimal specificity and sensitivity values for the tool, in order to compare the results with the previously cited study by Kawasumi and al. [10]. To do this, only the sinuses whose results were significant were used.

Considering the measures as independent variables, a logistic regression was also performed to study the influence of each variable on the separation of the groups (dependent variables). The logistic regression also yielded a prediction function allowing us to classify a case in one of the groups in the future. The logistic regression was preferred to a linear discriminant analysis because the conditions of use are more flexible. According to the results of the statistical tests carried out beforehand, the relevant variables were selected for the logistic regression, according to two different models. The training of the first model was carried out with the same data used for the test. As for the second model, the data was randomly divided into two groups, one used for training and the other for testing. A better result is expected for the first model since training and test data are identical.

Finally, in addition to this statistical analysis, the potentially problematic cases were visually inspected.

## Results

### Mean density (MD)

**Fig. 3:**
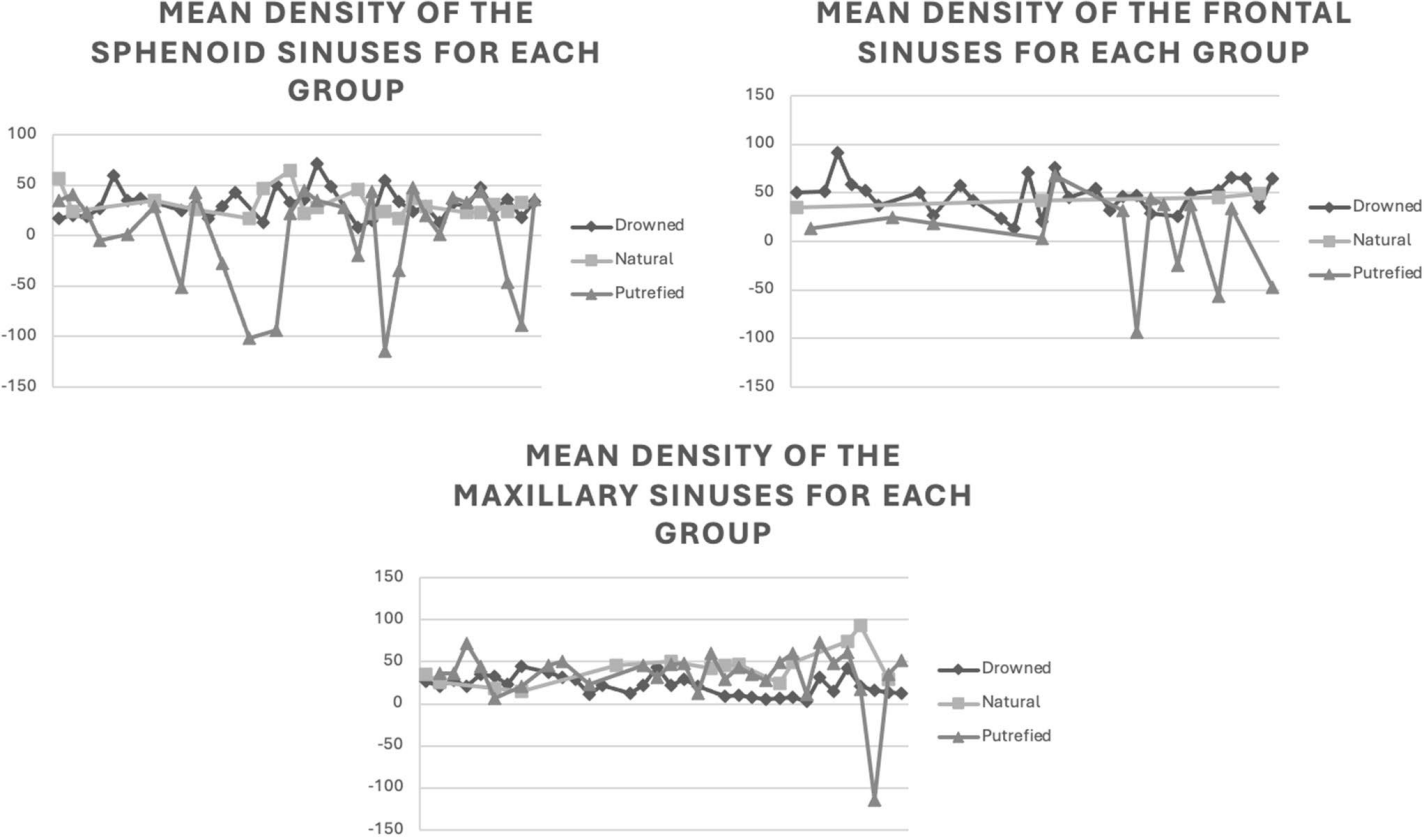
Mean CT densities of liquid (HU), by sinus for each group

For all sinuses, the MD measured in HU was significantly different between the putrefied and drowned groups (frontal sinuses *p* = 0.0008, maxillary sinuses *p* = 0.0002, sphenoid sinuses *p* = 0.0415) as depicted in Fig. 3. Between the group of natural death and the drowned group, the MD was significantly different for the maxillary sinus (*p* = 0.0005) but not for the frontal and sphenoid sinuses (respectively *p* = 0.3927 and *p* = 0.7691).

### Average liquid-ratio (LR)

The mean LR was statistically different for the frontal (*p* = 0.0003) and maxillary sinuses (*p* = 0.0004) when comparing the drowned and putrefied group. The mean LR was not different for the sphenoid sinuses (*p* = 0.2866). When comparing the natural death and the drowned group, a significant difference was found for all sinuses (frontal sinuses *p* = 0.0000, maxillary sinuses *p* = 0.0000 and sphenoid sinuses *p* = 0.0000).

### ROC curves for MD and LR

Using cut-off values ​​as Youden indices from ROC curves, promising specificities and sensitivities were obtained, using a single measurement (Table 1).

**Table 1:** Summary of results for ROC curves. Each entry indicates cutoff, area under the ROC curve (AUC), sensitivity (Se) and specificity (Sp)

Frontal sinus	Average liquid ratio	Cut-off 0.15 AUC 0.75 Se 68% et Sp 76%
	Mean density	Cut-off 44.55 HU AUC 0.82 Se 64% et Sp 93%
Maxillary sinus	Average liquid ratio	Cut-off 0.14 AUC 0.74 Se 86% et Sp 56%
	Mean density	Cut-off 34.91 HU AUC 0.78 Se 85% et Sp 65%
Sphenoid sinus	Average liquid ratio	Cut-off 0.17 AUC 0.57 Se 67% et Sp 50%
	Mean density	Cut-off 1.77 HU AUC 0.65 Se 100% et Sp 41%

Among all studied sinuses, the frontal sinuses have the highest area under the ROC curve (AUC) for both the amount of fluid and its density. When using only the MD, the choice of using the MD cut-off of a specific sinus depends on the desired specificity and sensitivity (e.g., use of the sphenoid sinus for high sensitivity or the frontal sinus for high specificity).

### Logistic regression

A logistic regression was performed (Excel extension « RegressItLogistic » (v. 2021.06.18)) in an attempt to classify the cases according to discriminant variables. Only the variables whose AUC ≥ 0.70 were considered. Indeed, the greater the value of the AUC, the more reliable the way of classifying the two groups. The MD and LR AUCs of the frontal and maxillary sinuses fell within this criterion. These were therefore the sinuses that were used for the average density and for the surface ratio. Indeed, the sphenoid sinuses did not show promising results, which is why logistic regression was not performed on them.

Three analyses were carried out in order to investigate the best possible combinations, namely: the combination of the average LR for the frontal and maxillary sinuses, the combination of the MD for the frontal and maxillary sinuses, and finally, the combination of the two characteristics. The results are reported in Table 2. However, it is important to mention that for each analysis, the used software only takes into account cases in which the variables of interest are fully available. Therefore, the sample size was decreased for each of them, for example when a sinus did not contain any liquid and therefore did not have any density measurement.

**Table 2:** Summary of results for logistic regression of frontal and maxillary sinuses when training and testing datasets are identical (a) or different (b)

1. Combination of mean densities	Model (a)	AUC 0.89 Se 92.6% et Sp 73.3%
	Model (b)	AUC 0.95 Se 90% et Sp 80%
2. Combination of mean densities and average liquid ratios	Model (a)	AUC 0.89 Se 92.6% et Sp 80%
	Model (b)	AUC 0.95 Se 95% et Sp 70%
3. Combination of average liquid ratios	Model (a)	AUC 0.74 Se 60.6% et Sp 70.6%
	Model (b)	AUC 0.74 Se 52% et Sp 79.3%

Models only based on the average LR performed the worst, in terms of AUC, sensitivity and specificity. The combination of MD from both sinuses resulted in better results, but the combination of MD and LR was the most successful. As expected, a decrease of the performance was observed when training and test datasets were different, although it appeared as reasonable.

## Discussion

First of all, the MD difference offers an initial estimate of the substance’s nature present in the liquid. In fact, the radiodensity of blood is between 40 and 60 HU while water’s is equal to 0 HU [14]. As for serous fluids, they are between 15 and 30 HU. In this sample, the cases of natural death and the cases of drowning had close values between 20 and 50 HU. The fluid could therefore contain blood, mixed with serous fluid. As for the average densities of the altered bodies, the values ​​were very variable for the various sinuses, ranging from 0 HU up to 35 HU at the most. However, the sample also had extreme negative (-127 HU) and positive (200 HU) values (Fig. 2). According to Zech and al. [14], putrefaction fluids show a range of HU values ​​between − 130 and 80 HU, explaining the negative values. As for the positive values, this could be explained by the presence of other liquids, such as cerebrospinal fluid or compounds resulting from cell degradation during post-mortem phenomena of autolysis and putrefaction, which add to the mixture and thus increase the density values.

Then, regarding the LR, statistical analysis in each sinus corroborates one of the conclusions drawn by Kawasumi and al. [8], stating that the presence of liquid is not specific to drowning, but the absence of liquid is specific to non-drowning. In other words, their study demonstrated that the presence of fluid accumulation in the maxillary or sphenoid sinuses on the CT-scan was significantly more common in drowning cases than in non-drowning cases. Another study, conducted by Lundemose and al. [15] also suggests that the presence of fluid in the frontal, maxillary and sphenoid sinuses could indicate drowning. Results also demonstrated a significantly different amount of fluid between the drowning group and the natural death group for the frontal (*p* = 0.0000), maxillary (*p* = 0.0000) and sphenoid (*p* = 0.0000) sinuses.

Finally, the combination of the MD and LR features improved the performance for model (a) for both sensitivity and specificity, but it was less conclusive for model (b). In general, performance metric values were as expected better with model (a) that used the same training and test datasets, but with a possible risk of overfitting.

**Fig. 4:**
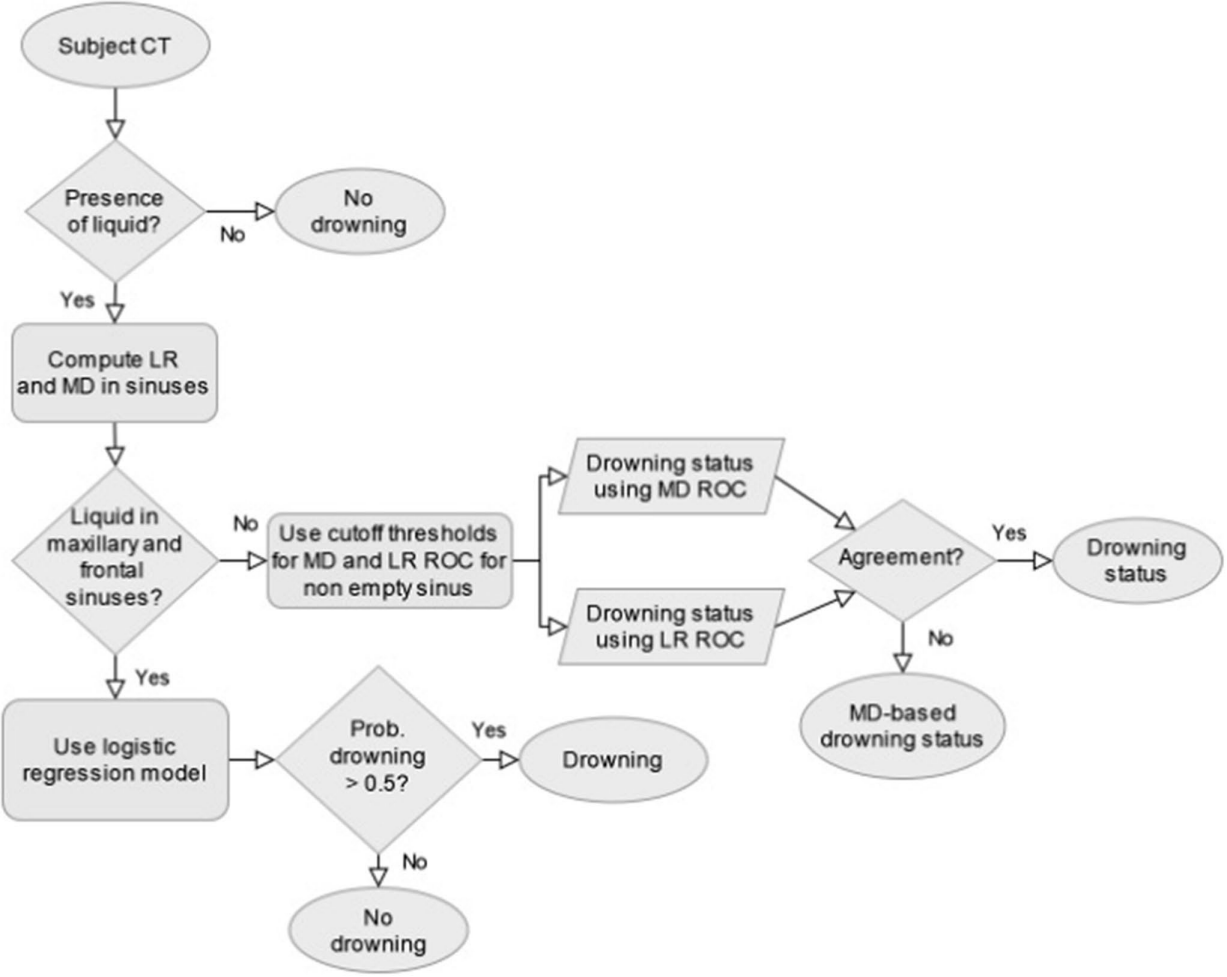
Decision algorithm for discrimination between groups (MD = mean HU density, LR = mean liquid ratio)

Based on our results, cut-off values for the frontal and maxillary sinuses can be proposed, as well as a step-by-step protocol for discrimination (Fig. 4).

In the case of death by drowning, it would therefore be necessary to check that all the sinuses contain liquid. If this is the case, the logistic regression equation of the model, based on the densities and surface ratios of the maxillary and frontal sinuses, is recommended. It can be admitted that if the probability produced by the logistic regression model is higher than 0.5, the cause of death was probably drowning. However, if one of the sinuses is empty, the use of ROC curves with their respective cut-off values would be more appropriate.

It is possible that, after analysis, the frontal sinus ROC curve for area ratios gives a “drowning group” result, while the frontal sinus ROC curve for density gives a “weakened body group” result. In this case, the ROC curve for density is considered more reliable because its AUC, specificity, and sensitivity were generally better.

### Strengths and limitations of the study

First of all, by relying on the RAI, the state of alteration of the corpses was quantified, resulting in a third group exclusively composed of putrefied bodies. This adds value to the study because, to date, no other study has made this differentiation. Indeed, previous studies [6–9] were limited to two groups (drowned, non-drowned) without considering the state of decomposition of the body, which often leads to an accumulation of liquid in the paranasal sinuses affecting the results. Additionally, decision models with good specificity and sensitivity are proposed.

However, as this was a study carried out in Switzerland, only fresh water drowning cases were included. Furthermore, as the prevalence of drowning is low in Switzerland, the sample size (108) is not very large. This represents 36 cases for each of the groups. In addition, some cases (cases without sinuses, empty sinuses, no sinus, etc.) could not be used during certain statistical analyzes (ROC, logistic regression) as the number of resulting cases was too low. Another limitation of this study is the intra- and inter-observer variability, which was not calculated. Two people carried out the contours so the subjectivity could be reduced. Moreover, an expert validated all the ROI to increase the scientific level of the study.

Given this low prevalence, all cases between the commissioning of the CT and 2020 were included. However, this led to a group with a non-homogeneous RAI, varying between 0 and 100. Consequently, four individuals of the drowning group were also altered bodies. Despite their low number, it is difficult to accurately assess their impact on our statistical tests as the overall data sample was not very large– possibly impacting the statistical power of the different approaches. To address this problem of sample size, it would been interesting to include in a future work the cases of the Lausanne CURML site.

## Conclusion

In this study, a decision algorithm was introduced that leverages post-mortem CT measurements to distinguish drowned bodies. Specifically, this approach differentiates between bodies that have drowned, those undergoing advanced putrefaction, and cases of natural deaths with typical post-mortem changes. These findings support the further exploration and refinement of drowning diagnosis using post-mortem CT techniques.

## Data Availability

No.
